# INNOVATIONS IN EMERGENCY MEDICAL CARE: EARLY ADOPTERS OF GERIATRIC EMERGENCY DEPARTMENTS

**DOI:** 10.1093/geroni/igz038.1680

**Published:** 2019-11-08

**Authors:** John G Schumacher

**Affiliations:** University of Maryland, Baltimore County (UMBC), Baltimore, Maryland, United States

## Abstract

Hospital emergency departments (EDs) treat more than 20 million older adults each year making it a remarkably significant site of healthcare delivery. To date, the nation’s nearly 5,000 EDs have been slow to modify their staffing, ED training, procedures, or physical environment to better serve the unique needs our heterogeneous older adult population. Nonetheless, nationwide a small set of innovative EDs have redesigned their care and now promote themselves as Geriatric Emergency Departments (GEDs) which specifically tailor care to older adults and their families. Using a systematic, nationwide search process of U.S. hospitals, this research identified n=83 EDs which clearly self-identified as GEDs. All eligible GEDs were contacted with n=54 (65%) responding to our self-administered survey regarding their organization, delivery of care, and adherence to national guidelines on the emergency medical care of older adults. Results document a wide variety of care models, staffing patterns, screening procedures, clinical care modifications, quality improvement efforts, physical environment enhancements, referral patterns, and tracking of older patient outcomes. Analysis of open-ended responses demonstrated widely divergent interpretations of the national guidelines on emergency care for older adults including the definition of a GED. Based on the findings, research recommendations are made to researchers regarding the conceptualization and specific wording of future survey items in order to increase the reliability and validity of research into GEDs.

